# Efficacy of perioperative cryotherapy combined with intra-articular injection of tranexamic acid in total knee arthroplasty

**DOI:** 10.1097/MD.0000000000034381

**Published:** 2023-07-21

**Authors:** Xiao Huang, Fulin Li, Weifa Shi, Wenhui Liu, Wenwen Huang, Dong Yin

**Affiliations:** a Department of Joint Surgery and Sports Medicine, The People’s Hospital of Guangxi Zhuang Autonomous Region, Nanning, China.

**Keywords:** complications, cryotherapy, total knee arthroplasty, tranexamic acid

## Abstract

To evaluate the efficacy and safety of perioperative cryotherapy combined with intra-articular injection of tranexamic acid (TXA) in total knee arthroplasty (TKA) and explore a new strategy of enhanced recovery after TKA.

**Methods:**

We randomly divided 200 patients into 4 groups: normal saline (10 mL) by drainage (Group A, placebo); intra-articular injection of TXA (1 g, 10 mL, Group B); normal saline (10 mL) and continuous cryotherapy postoperatively (Group C) and intra-articular injection of TXA (1 g, 10 mL) and continuous cryotherapy postoperatively (Group D). Primary outcomes were blood loss volume, postoperative pain and circumference variation. We also recorded consumption of analgesics, postoperative length of stay (p-LOS), range of motion (ROM), function score (Hospital for Special Surgery) and severe complications.

**Results:**

There were statistically significant differences in postoperative drainage volume, total blood loss, hidden blood loss, and visual analogue scale at rest and walking on postoperative day 1 (POD1), POD2, POD3, ROM (POD3, 7, discharge, postoperative month), circumference variation (POD3, 7), p-LOS, Hospital for Special Surgery score (discharge) and drop of hemoglobin on POD3 (*P* < .05) among 4 groups, but there were no significant differences in intraoperative blood loss, postoperative prothrombin, activated partial thromboplastin time, overall number of patients or total consumption of oxycodone and perioperative complications (e.g., incidence of surgical site infection, deep venous thrombosis, and cold injury) among them (*P* > .05).

**Conclusion:**

Continuous cryotherapy combined with intra-articular injection of TXA provides short-term advantages in reducing blood loss, pain, postoperative swelling, p-LOS and increasing ROM and joint function in the early postoperative period after TKA without increasing any severe complications.

## 1. Introduction

Total knee arthroplasty (TKA) is recognized as an excellent surgical procedure for patients with end-stage knee osteoarthritis.^[1]^ However, there are some potential unresolved problems such as perioperative blood loss, incision pain, swelling of knee joint and limited range of motion (ROM) after TKA.^[2]^ Some patients with large blood loss volume (BLV) may lead to transfusion due to extensive soft tissue injury and osteotomy in TKA. Estimated blood loss, reported up to 1500 mL during perioperative period, may delay rehabilitation^[3]^ as the same as those problems including incision pain, knee swelling, limited ROM especially in the elderly patients.

Intravenous tranexamic acid (TXA) has been shown that can significantly reduce blood loss in total hip and knee arthroplasty.^[4,5]^ Its usage has been recommended in the guideline issued by the American Association of Hip and Knee Surgeons in 2019.^[6]^ However, it is still controversial to provide TXA with ideal method.^[7]^ Studies commonly believe that only a small percentage of TXA injected intravenously reaches the target location and may lead to impaired coagulation function because of repeated use by intravenous injection.^[8]^ Thus, it was reported that the result of a new method about TXA (intra-articular retrograde injection via draining) was satisfying, and that was associated with significant improvement in postoperative hemoglobin (Hb) decrease without systemic hypercoagulability.^[9]^ Besides, some literatures suggest that TXA may have a certain anti-inflammatory effect, which can reduce perioperative pain in TKA.^[10]^ However, it may cause some mild or transient side effects including deep vein thrombosis (DVT), nausea, vomiting, and dizziness with high-dose use of TXA according to the clinical application.^[11]^

There are several other strategies to reduce BLV during perioperative period, such as cryotherapy, draining clamping and different knee position.^[12,13]^ Cryotherapy was reported that people used ice or snow to reduce swelling and pain about 400 years before Christ by Hippocrates.^[14]^ It has a common basic principle that includes ice, rest, compression and elevation after acute soft tissue injury in the field of sport medicine. A large number of literatures have reported that patients with acute soft tissue injury have been effectively treated after cryotherapy,^[15]^ and some studies show postoperative cryotherapy in TKA was an effective approach to reduce postoperative blood loss, transfusion, pain, less swelling, and improve ROM.^[16,17]^

However, there are few reports of cryotherapy combined with TXA in TKA, especially continuous cryotherapy. Considering literature reports on the related effects of TXA and cold therapy, it is necessary to explore the synergistic effect of combined application. In this study, our aim is to evaluate the efficacy and safety of continuous cryotherapy combined with TXA in the perioperative period of TKA and explore a new strategy of enhanced recovery after TKA.

## 2. Materials and methods

This prospective, double-blind, randomized, controlled study was conducted from February 2017 to July 2022. Recruitment was approved by the institutional review board and registered at the Chinese Clinical Trial Registry (ChiCTR2300071161). All patients provided written informed consent before participation. Inclusion criteria were unilateral TKA for osteoarthritis and a signed informed consent form. Exclusion criteria were as follows: allergy for TXA and cold; age ≤ 50 years or ≥80 years; active phlebitis and diabetes with vascular lesion; glucocorticoid use within 3 months or any strong opioids and anticoagulants within a week; history of severe heart disease (NYHA > 2), liver/kidney failure, or systemic rheumatic diseases (rheumatoid arthritis, ankylosing spondylitis, systemic lupus erythematosus); DVT, pulmonary embolism, and coagulopathy before surgery; prior ipsilateral knee surgery; lack of cognitive function or normal sensation; preoperative anemia; and loss to follow-up.

The 200 patients were randomly divided into 4 groups (A, B, C, and D), each with 50 patients. All patients were randomly assigned sequences hidden in opaque, sealed envelopes that were opened before surgery. All patients receive intravenous TXA (1 g, 100 mL normal saline, NS) 30 minutes before releasing tourniquet and the drainage was clamped for the first 4 hours after operation. Patients in Group A received an intra-articular injection of NS (10 mL) by drainage and then clamped. Patients in Group B received an intra-articular injection of TXA (1 g, 10 mL NS) by drainage and then clamped. Patients in Group C received an intra-articular injection of NS (10 mL) by drainage and then clamped and continuous cryotherapy postoperatively. Patients in Group D received an intra-articular injection of TXA (1 g, 10 mL NS) by drainage and then clamped and continuous cryotherapy postoperatively. The continuous cold flow device adopted was Evercryo (China). Cryotherapy continues to perform 90 minutes per 120 minutes until 7 days after surgery and ice was changed in the canisters every 4 hours by the nursing staff. All participants, surgeons, anesthesiologists, nurses, and research assistants were blinded to group allocation.

### 2.1. Patient demographics

**Figure 1.**
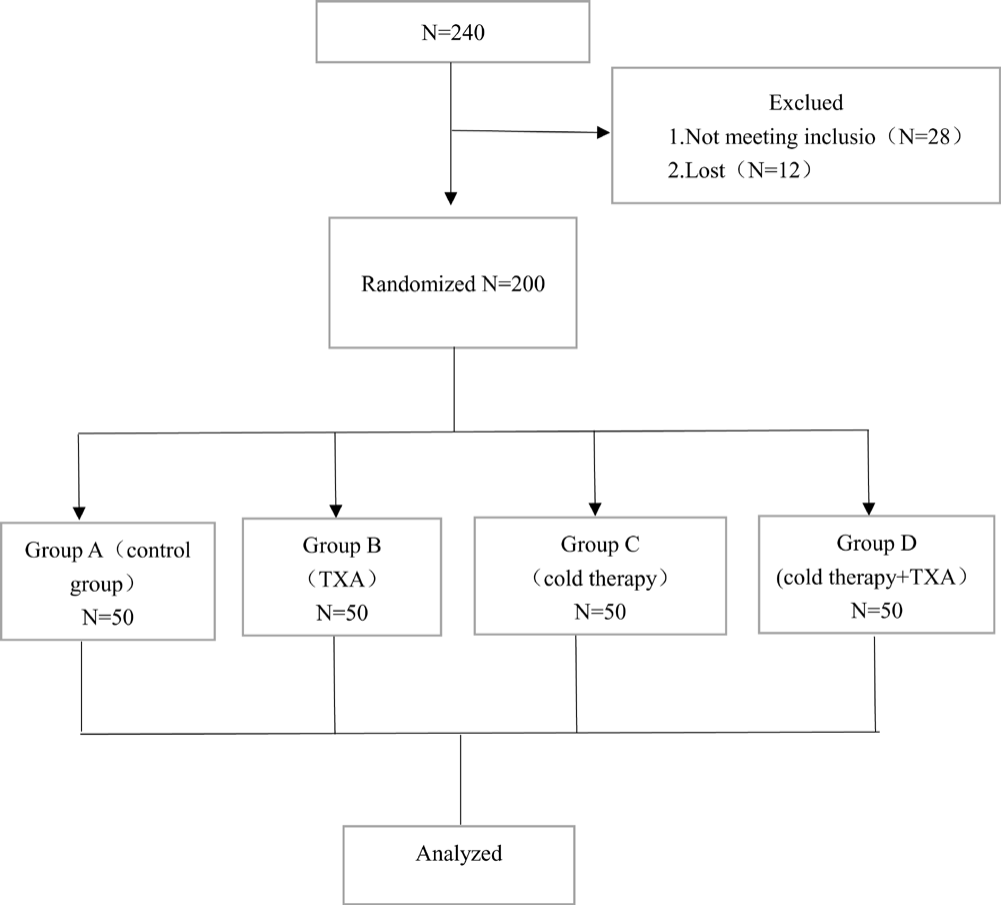
Schematic diagram of the patient study process.

From February 2017 to July 2022, 240 patients scheduled to undergo primary unilateral TKA were screened for inclusion in the study. Twenty-eight patients did not meet the inclusion criteria, and twelve patients were lost to follow-up at the 3-month endpoint, leading to 200 participants (Fig. 1). There was no statistical difference in the general data of the patients (Table 1).

**Table 1:** Demographic data of the patients receiving TKA.

	Group A	Group B	Group C	Group D	*P*
N	50	50	50	50	–
Age (yr)	64.56 ± 6.00	66.52 ± 7.20	65.78 ± 7.04	65.92 ± 6.85	.59
Gender (M/F)	16/34	15/35	15/35	17/33	.97
BMI (kg/m^2^)	25.59 ± 3.37	25.56 ± 3.39	24.93 ± 3.15	25.33 ± 3.66	.77
Hypertension (Y/N)	17/33	16/34	17/33	15/35	.97
Preoperative PT (s)	13.09 ± 0.55	13.11 ± 0.58	12.94 ± 0.38	12.98 ± 0.45	.22
Preoperative APTT (s)	36.80 ± 1.79	36.48 ± 1.76	36.67 ± 1.59	36.51 ± 1.66	.57
Preoperative Hb (g/L)	126.43 ± 8.89	124.52 ± 8.16	125.88 ± 7.56	125.22 ± 7.69	.62
Preoperative HSS score	38.70 ± 3.24	40.22 ± 4.71	39.98 ± 4.36	40.08 ± 4.80	.27
Preoperative VAS	7.18 ± 0.80	7.34 ± 0.72	7.32 ± 0.71	7.24 ± 0.80	.70
Operating time (min)	128.26 ± 19.68	125.32 ± 18.85	123.40 ± 14.17	124.96 ± 17.72	.58

APTT = activated partial thromboplastin time, BMI = body mass index, Hb = hemoglobin, HSS = Hospital for Special Surgery, PT = postoperative prothrombin, TKA = total knee arthroplasty, VAS = visual analogue scale.

All surgeries were performed by a senior physician of the surgical team in a hundred-level laminar flow operating room. Patients were evaluated by an anesthesiologist and given intraspinal anesthesia in the supine position, middle approach, and bone cement prosthesis (posterior stabilized; DePuy). DVT was detected by lower extremity doppler ultrasound in all patients before discharge.

### 2.2. Postoperative management

Ankle dorsal, plantar flexion, and quadriceps strength exercises began in the recovery bay. These 4 regions would receive rivaroxaban (10 mg) orally when the drainage was removed at the first 48 postoperative hours. Patients received standard supervised physiotherapy daily, including continuous passive motion, strength training, and walking. After returning to the ward, pain was assessed using a visual analog scale (VAS; 0: no pain, 10: the worst pain imaginable). If VAS scores were between 4 and 6, oxycodone was administered orally at Q8h (10 mg). If pain VAS exceeded 6, 40 mg of Parecoxib (Pfizer, NY) was administered intravenously. The transfusion protocol trigger, according to the agreement which is provided by National Health Commission of People’s Republic of China, was <70 g/L measured at 24 and 72 hours postoperatively.

### 2.3. Outcome assessment

Intraoperative blood loss, postoperative drainage volume (PDV), VAS at rest and walking on postoperative day 1 (POD1), POD2, POD3, ROM (POD3, 7, discharge, postoperative month), circumference variation (POD3, 7), postoperative length of stay (p-LOS), function score (Hospital for Special Surgery [HSS], discharge) and drop of Hb on POD3 were recorded. Total number and dose of oxycodone, postoperative prothrombin, activated partial thromboplastin time, and perioperative complications (surgical site infection [SSI], DVT, cold injury) were also noted. The total blood loss (TBL) and hidden blood loss (HBL) were calculated by the methods of Nadler.^[18]^

### 2.4. Statistical analysis

Statistical analyses were performed in SPSS version 24 (SPSS Inc., Chicago, IL). Data are presented as means ± standard deviation (continuous variables) or raw numbers (qualitative variables). One-way ANOVA and Tukey’s post hoc test were used to evaluate parametric data, while the Mann–Whitney *U* test was used for nonparametric data. Qualitative comparative data were analyzed with Pearson’s chi-square test or Fisher’s exact test. Significance was set at *P* < .05. With the power of 0.90 and the significant level of 0.05, 43 patients per arm were required in the study. Considering the dropping out, 10% to 15% of the sample size should be increased. Therefore, the sample size of 50 cases was required for each group, so the total sample size was 200 cases.

## 3. Results

### 3.1. Primary outcomes

#### 3.1.1. Blood loss volume.

**Figure 2.**
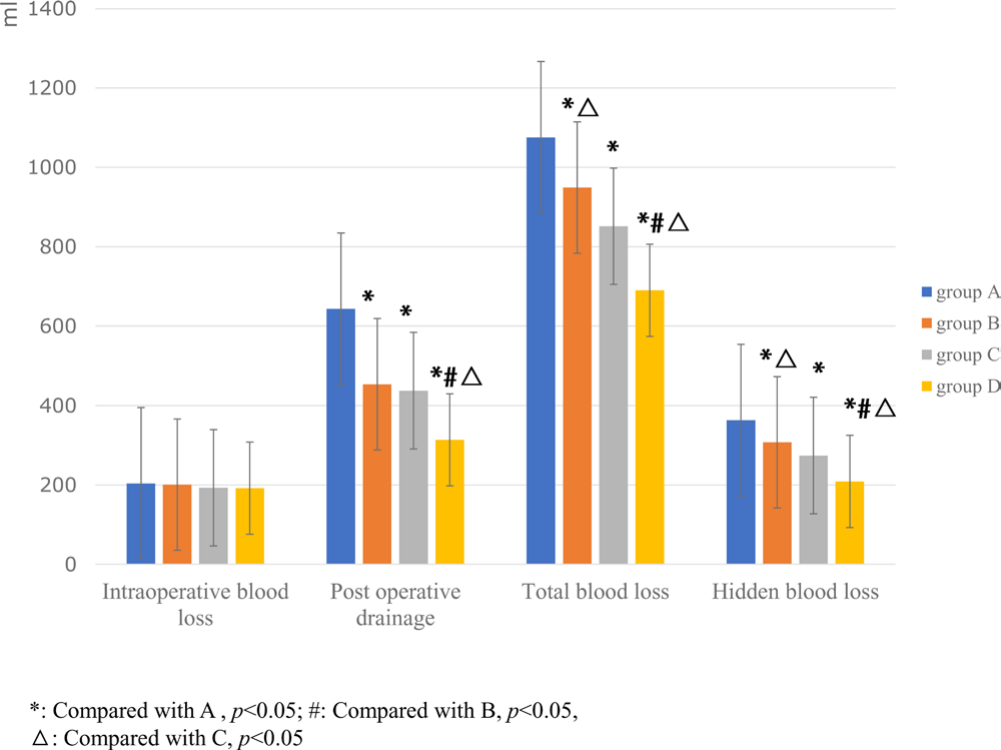
The comparison of blood loss among the 4 groups. The one-way ANOVA of variance was performed to detect the difference among the groups (*P < .05).

The PDV, TBL, HBL in Groups B (453.60 ± 52.84; 949.06 ± 224.94; 307.60 ± 83.34), C (437.50 ± 54.38; 851.40 ± 104.71; 274.06 ± 56.12), and D (313.76 ± 60.3; 689.96 ± 100.40; 208.86 ± 67.89) were generally lower than those in Group A (636.60 ± 99.13; 1075.68 ± 212.59; 363.02 ± 93.98) (*P* < .05). Comparison between any 2 groups, there were statistically significant differences in PDV, TBL and HBL among the 4 groups (*P* < .05), except the PDL between Group B and C (*P* = .25) (Fig. 2).

**Figure 3.**
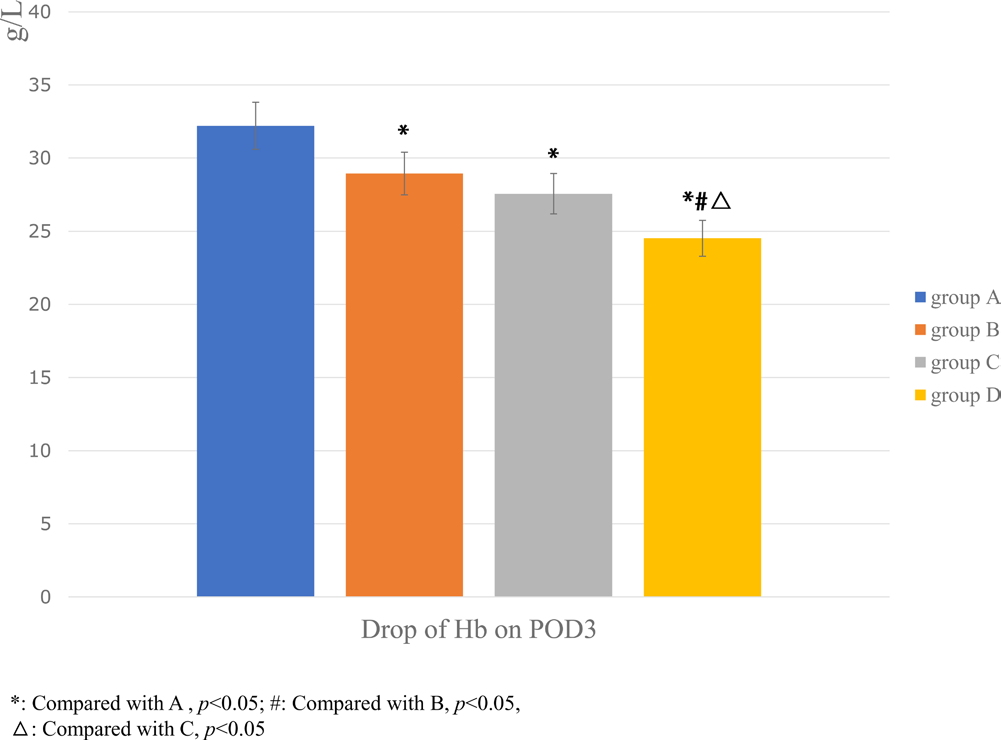
The comparison of drop of Hb on POD3 among the 4 groups. The one-way ANOVA was performed to detect the difference between the groups (*P < .05). Hb = hemoglobin, POD = postoperative day.

The drop of Hb on POD3 in Group B (28.94 ± 6.66), C (27.56 ± 5.12), and D (24.52 ± 5.01) were generally lower than that in Group A (32.20 ± 3.11) (*P* < .05). Comparison between any 2 groups, there were statistically significant differences in drop of Hb on POD3 among the 4 groups (*P* < .05) (Fig. 3).

There was no statistically significant difference among the 4 groups in intraoperative blood loss (*P* = .79) (Fig. 3).

#### 3.1.2. Pain.

**Figure 4.**
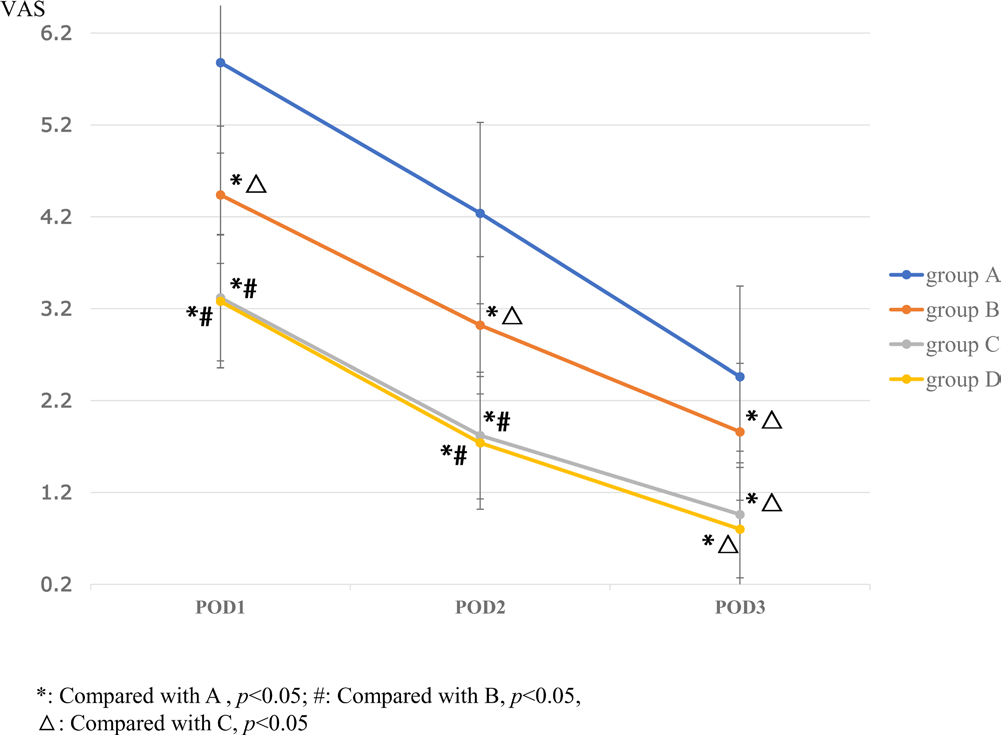
The comparison of VAS of pain at rest among the 4 groups on POD 1, 2, and 3. The one-way ANOVA was performed to detect the difference among the groups (*P < .05). POD = postoperative day, VAS = visual analogue scale.

On POD1, 2 and 3, pain scores at rest were significantly lower for Group B (4.44 ± 0.58; 3.02 ± 0.71; 1.86 ± 0.67), C (3.32 ± 0.84; 1.82 ± 0.69; 0.96 ± 0.70), and D (3.28 ± 0.81; 1.74 ± 0.57; 0.80 ± 0.54) than for Group A (5.58 ± 0.61; 4.24 ± 0.70; 4.44 ± 0.58) (*P* < .05). Comparison between any 2 groups, there were statistically significant differences in POD1, 2 and 3 among the 4 groups (*P* < .05), except between Group C and D (*P* = .78; *P* = .57; *P* = .20) (Fig. 4).

**Figure 5.**
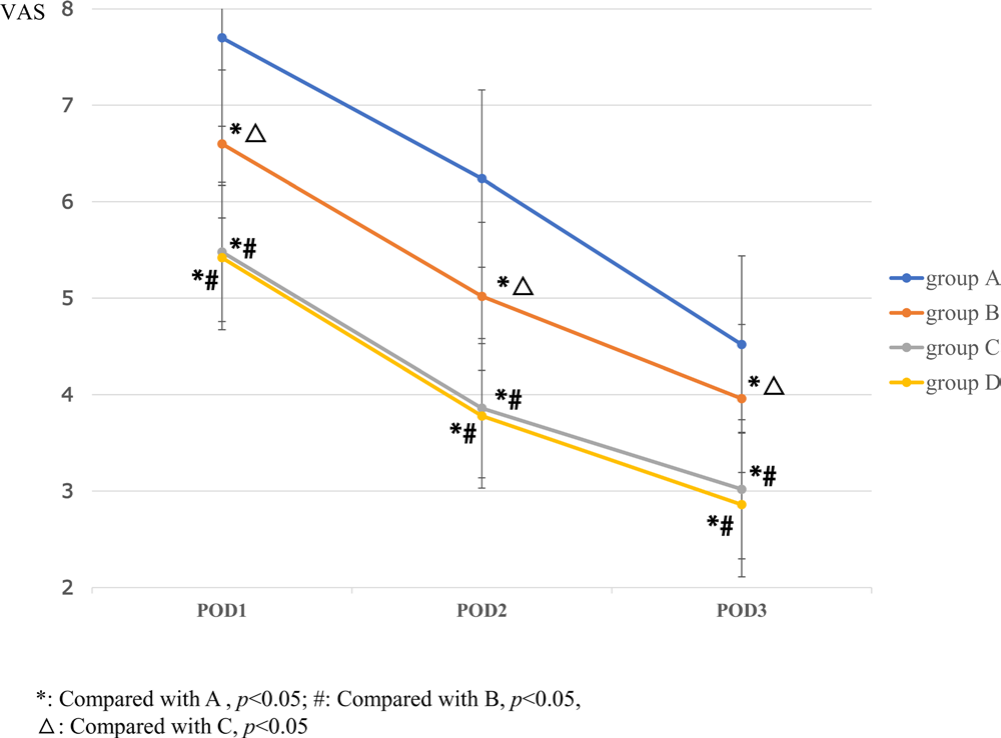
The comparison of VAS of pain at walking among the 4 groups on POD 1, 2, and 3. The one-way ANOVA was performed to detect the difference among the groups (*P < .05). POD = postoperative day, VAS = visual analogue scale.

Consistent with pain scores at rest on POD1, 2 and 3, dynamic pain scores on POD1, 2, and 3 were significantly lower for Group B (6.60 ± 0.61; 5.02 ± 0.69; 3.96 ± 0.70), C (5.48 ± 0.89; 3.86 ± 0.73; 3.02 ± 0.65), and D (5.42 ± 0.84; 3.78 ± 0.62; 2.86 ± 0.50) than for Group A (7.70 ± 0.54; 6.24 ± 0.74; 4.52 ± 0.58) (*P* < .05). Comparison between any 2 groups, there were statistically significant differences in POD1, 2 and 3 among the 4 groups (*P* < .05), except between Group C and D (*P* = .68; *P* = .57; *P* = .19) (Fig. 5).

#### 3.1.3. Joint swelling.

Circumference variation on POD3 and 7 were significantly lower for Group C (2.82 ± 0.44; 1.44 ± 0.46) and D (2.70 ± 0.27; 1.49 ± 0.36) than for Group A (4.25 ± 0.42; 4.01 ± 0.30) and B (4.23 ± 0.40; 3.90 ± 0.40) (*P* < .05). There was no statistically significant difference between Group A and B (*P* = .82; *P* = .16) or Group C and D (*P* = .12; *P* = .53) on POD3 and 7 (Table 2).

**Table 2:** The clinical effect among the 4 groups.

	Group A	Group B	Group C	Group D	*P*
Difference in circumference on POD3 (cm)	4.25 ± 0.42	4.23 ± 0.40	2.82 ± 0.44	2.70 ± 0.27	.00
Difference in circumference on POD3 (cm)	4.00 ± 0.30	3.90 ± 0.40	1.44 ± 0.46	1.49 ± 0.36	.00
HSS score at discharge	79.98 ± 4.48	80.40 ± 3.27	86.58 ± 4.70	85.94 ± 3.86	.001
Oxycodone					
N	36/50	37/50	33/50	34/50	.81
Total dose (mg)	730	580	530	560	.28
P-LOS	12.90 ± 1.33	12.08 ± 1.41	10.76 ± 1.17	10.46 ± 1.53	.00

HSS = Hospital for Special Surgery, POD = postoperative day, P-LOS = postoperative length of stay.

### 3.2. Secondary outcomes

#### 3.2.1. Range of motion.

**Figure 6.**
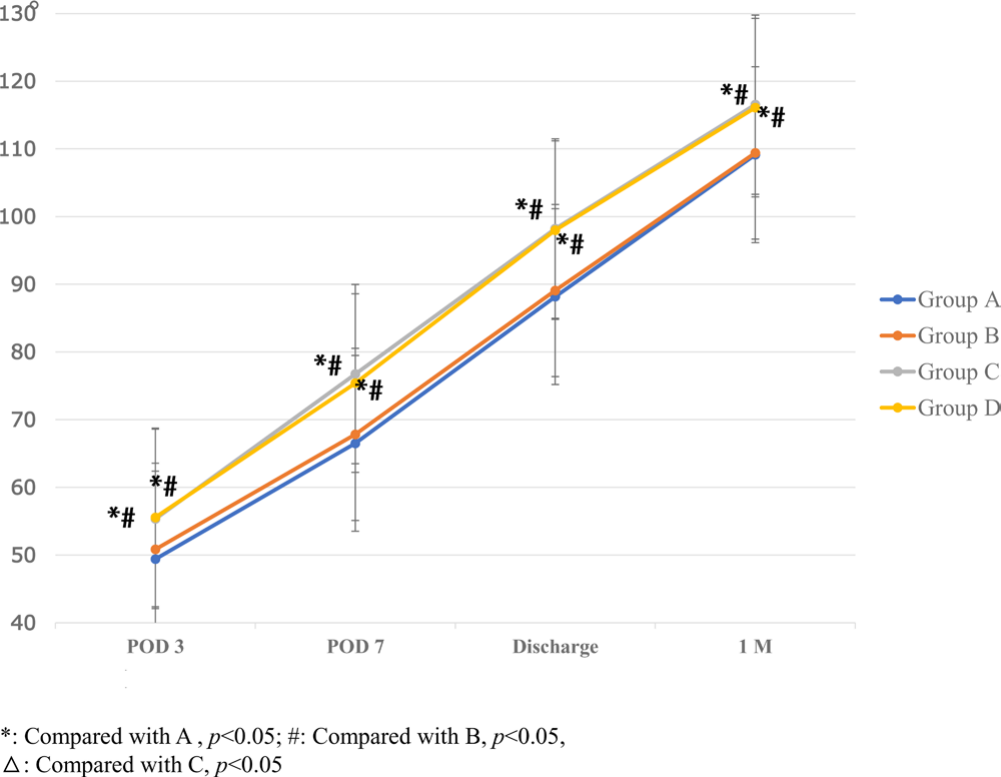
The comparison of ROM among the 4 groups on POD 3, 7, discharge and 1M. The one-way ANOVA was performed to detect the difference among the groups (*P < .05). POD = postoperative day, ROM = range of motion.

ROM on POD3, 7, discharge and 1M were significantly greater for Group C (55.34 ± 6.35; 76.74 ± 8.47; 98.22 ± 6.95; 116.54 ± 4.54) and D (55.36 ± 3.98; 75.40 ± 6.42; 98.00 ± 5.36; 116.10 ± 4.73) than for Group A (49.40 ± 4.14; 66.50 ± 3.35; 88.18 ± 3.85; 109.14 ± 2.41) and B (50.84 ± 4.44; 67.82 ± 2.88; 89.08 ± 2.95; 109.40 ± 2.93) (*P* < .05). There was no statistically significant difference between Group A and B (*P* = .14; *P* = .25; *P* = .37; *P* = .73) or Group C and D (*P* = .82; *P* = .25; *P* = .83; *P* = .56) on POD3, 7, discharge and 1M (Fig. 6).

#### 3.2.2. Postoperative length of stay and knee function.

P-LOS were significantly less for Group B (12.08 ± 1.41), C (10.76 ± 1.17), and D (10.46 ± 1.53) than for Group A (12.90 ± 1.33) (*P* < .05), also Group B and C (*P* < .05) or Group B and C (*P* < .05), but there was no statistically significant difference between Group C and D (*P* = .27).

HSS score at discharge were significantly higher for Group C (86.58 ± 4.70) and D (85.94 ± 3.86) than for Group A (79.98 ± 4.48) and B (80.40 ± 3.21) (*P* < .05). There was no statistically significant difference between Group A and B (*P* = .61) or Group C and D (*P* = .44).

#### 3.2.3. Oxycodone consumption, coagulation function and complications.

There was no statistically significant difference in total number and dose of oxycodone for Group A (36/50; 730 mg), B (37/50; 580), C (33/50; 530) and D (34/50; 560) among the 4 groups (*P* = .81; *P* = .28) (Table 2), and no significant difference in prothrombin (*P* = .20) and activated partial thromboplastin time (*P* = .75) (Table 3).

**Table 3:** The postoperative coagulation and complications.

	Group A	Group B	Group C	Group D	*P*
PT	13.07 ± 0.30	13.13 ± 0.55	13.19 ± 0.54	12.99 ± 0.53	.20
APTT	36.50 ± 1.76	36.19 ± 1.84	36.23 ± 1.81	36.15 ± 1.66	.75
DVT	1/50	2/50	2/50	1/50	.89
Cold injury	0	0	0	0	–
SSI	0	0	0	0	–

APTT = activated partial thromboplastin time, DVT = deep venous thrombosis, PT = postoperative prothrombin, SSI = surgical site infection.

The complication rate of DVT for Group A, B, C and D was 1/50, 2/50, 2/50 and 1/50, and there was no statistically significant difference (χ^2^ = 0.62; *P* = .89) (Table 3). In this study, we did not find any SSI or frostbite incidents.

## 4. Discussion

Cryotherapy, also known as cold therapy, as an age-old treatment method on acute soft tissue injury, is gradually applied to TKA postoperative rehabilitation, which is also utilized to enhance recovery and outcomes after TKA.^[19]^ Despite various advances in postoperative rehabilitation, cryotherapy remains popular and is generally considered attractive because of its minimal disadvantages compared with the possible benefits.^[19]^ External application of cryotherapy in TKA is supposed to reduce the intra-articular temperature, which on the one hand, could slow the conduction velocity of nerve fibers and then probably reduce pain transmission, and on the other hand, could reduce peripheral blood flow due to circulating vasoconstriction and therefore decrease the local inflammation and swelling.^[19]^ However, traditional ice bag or gel pack is more common, which is typically discontinuous with unregulated cold temperature and demands a manual replacement by the nurses. As a new kind of cryotherapy, continuous pressurized cryotherapy, developed to deliver a steady cooling temperature for a prolonged time,^[20]^ are relatively rare reported, and combined with intra-articular injection of tranexamic acid literature have not been reported. In our study, we found continuous cryotherapy combined with intra-articular injection of TXA provides short-term advantages in reducing BLV, pain, postoperative swelling, p-LOS and increasing ROM and joint function in the early postoperative period after TKA without increasing any severe complications.

How to further reduce perioperative blood loss of TKA is the point we have been working on. TXA has been well documented in reducing blood loss, whether intravenously, topically, or orally,^[5]^ but there does not seem to be a consensus on this aspect of cryotherapy. The efficacy of cryotherapy in terms of blood loss has been reported relatively infrequently and is often used as a secondary goal. A total of 78 patients were included in a prospective randomized controlled study by Bech et al.^[21]^ The results showed that Hb decreased by 7.7 g/L in the experimental group and 8.7 g/L in the control group on POD3, with no statistically significant difference (*P* = .68). Adie et al^[22]^ concluded through the meta-analysis of randomized controlled studies that cold therapy could effectively reduce perioperative blood loss (*P* = .01), but could not reduce the transfusion rate (*P* = .17). In our study, as the primary study outcome, we found that cryotherapy combined with intra-articular injection of TXA could effectively reduce PDV, TBL, HBL and drop of Hb on POD3. On one hand, we believe that pressure cryotherapy can play a role of compression, increasing hydrostatic pressure to reduce the flow of fluid in the interstitium and reduce exudation. At the same time, it can reduce the volume of joint cavity, reduce blood accumulation, increase the pressure in the joint cavity, reduce local capillary bleeding, and then play a hemostatic role. On the other hand, cryotherapy can stimulate skin cold receptors, which can cause blood vessels to contract and then reduce bleeding. In addition, by comparing Group A and B or Group A and C, we found that TXA or cold therapy has a good hemostatic effect, which is consistent with previous studies.^[9,13]^ By comparing Group B and C, we found that the new type of cryotherapy was more effective in reducing the HBL and TBL than the simple articular injection of TXA, but there was no statistical difference in the PBV (*P* = .24) and drop of Hb on POD3 (*P* = .25). We believe that this is because of the short half-life of TXA, while continuous cryotherapy reduces HBL and therefore TBL due to the longer cycle pressure.

Perioperative pain and joint swelling of TKA, which have been affecting patients’ satisfaction, were also the primary outcomes of this study. Some studies have proved that cryotherapy can reduce perioperative joint pain, swelling, improve ROM and shorten p-LOS. In an earlier prospective randomized controlled study, Sadoghi et al^[12]^ reported that the new pressor cryotherapy devices were more effective than conventional ones in reducing postoperative pain and inflammation. In a prospective randomized controlled study, Liao and Xu^[23]^ demonstrated that a new cryotherapy combined with accelerated rehabilitation strategies can improve the postoperative swelling and pain, inspire patients’ autonomy in postoperative rehabilitation training, shorten the time to out-of-bed activity, promote recovery of knee function, and improve patients’ postoperative self-care ability and quality of life. However, there are also some studies suggest that the new type of cryotherapy has no significant advantage over traditional ice. In a prospective randomized controlled study, compared with traditional ice, Bech et al^[21]^ found no additional benefit of consistent cryotherapy on postoperative pain, ROM, nausea or vomiting, opioid use, blood loss, lower limb function, or length of stay. But, in any case, current research supports the idea that cryotherapy can effectively reduce joint pain and swelling, which is consistent with our research. In this study, compared with Group B, Group D was superior to VAS at rest and walking on POD1, 2, 3, ROM (POD3, 7, discharge, postoperative month), circumference variation (POD3, 7), p-LOS, HSS score (discharge) and drop of Hb on POD3. It should be noted that there was no statistically significant difference in total number and dose of oxycodone among the 4 groups (*P* = .81; *P* = .28), which may be related to the continuous use of parecoxib after surgery. Therefore, we can conclude that the new cryotherapy can reduce the perioperative pain and swelling of TKA, improve the ROM, and accelerate postoperative rehabilitation. In view of this result, we considered external application of cryotherapy in TKA can reduce the intra-articular temperature, which can slow the conduction velocity of nerve fibers and then probably reduce pain transmission and reduce peripheral blood flow due to circulating vasoconstriction and therefore decrease the local inflammation and swelling. Although some studies have reported that TXA can reduce inflammation and relieve pain, it has not been confirmed in our study,^[24,25]^ which might be related to small dose. By comparing Group C and D, we concluded that the new cryotherapy combined with intra-articular injection of TXA had no advantage except in reducing blood loss, which may also confirm that cryotherapy combined with TAX has no synergistic effect on reducing inflammation. However, by comparing the VAS of Group A and B at rest and walking on POD1, 2, 3, we found that TXA has some analgesic effect, which may mediately confirm the anti-inflammatory effect of TXA? At the same time, by comparing the VAS of Group B and C at rest and walking on POD1, 2, 3, we found that the new cryotherapy has more advantages in pain relief than TXA.

For new cryotherapy combined with intra-articular injection of TXA, it is generally believed that the incidence of complications is more concern. In this study, the incidence of DVT in Group A, B, C and D was 1/50, 2/50, 2/50 and 1/50, and there was no significant difference in the incidence of perioperative DVT among the 4 groups (*P* = .89), which is the same as previous studies.^[17,23,25]^ Besides, SSI and frostbite are also complications that we cannot ignore. There were not any SSI or cold injury in this study, which confirmed the safety of the new cryotherapy combined with intra-articular injection of TXA.

This study had some limitations. Firstly, 3 months of follow-up might have concealed different long-term safety profiles in our study. Secondly, the small sample and single center study may weaken the convincing power of our study. Thirdly, this study cannot confirm whether continuous cold flow device is superior to traditional cryotherapy, which is what we need to work on next. Fourthly, single and small doses of TXA may have attenuated the anti-inflammatory effect, and we may try multiple and large doses for further confirmation. Fifthly, we explored the effect of continue cryotherapy combined with TXA without involving clear temperature and suitable dose of TXA for the best effect, and our further study might perform several studies to explore the effect of different temperatures combined with different doses of TXA.

## 5. Conclusion

Continuous cryotherapy combined with intra-articular injection of TXA provides short-term advantages in reducing blood loss, pain, postoperative swelling, p-LOS and increasing ROM and joint function in the early postoperative period after TKA without increasing any severe complications.

## Author contributions

**Conceptualization:** Dong Yin.

**Data curation:** Xiao Huang, Fulin Li, Weifa Shi, Wenhui Liu, Wenwen Huang.

**Formal analysis:** Xiao Huang, Fulin Li.

**Funding acquisition:** Xiao Huang.

**Investigation:** Xiao Huang.

**Methodology:** Xiao Huang.

**Project administration:** Xiao Huang, Dong Yin.

**Resources:** Wenhui Liu.

**Software:** Weifa Shi, Wenhui Liu, Wenwen Huang.

**Supervision:** Wenwen Huang.

**Writing – original draft:** Fulin Li, Weifa Shi, Dong Yin.

**Writing – review & editing:** Fulin Li, Dong Yin.
